# Preoperative halo-gravity traction with and without thoracoscopic anterior release for skeletal dysplasia patients with severe kyphoscoliosis

**DOI:** 10.1007/s11832-016-0721-0

**Published:** 2016-03-26

**Authors:** Sina Pourtaheri, Suken A. Shah, Colleen P. Ditro, Laurens Holmes, William G. Mackenzie

**Affiliations:** Department of Orthopaedics, Nemours/Alfred I. duPont Hospital for Children, P.O. Box 269, Wilmington, DE 19899 USA

**Keywords:** Skeletal dysplasia, Kyphoscoliosis, Halo-gravity traction, Scoliosis

## Abstract

**Purpose:**

Recent work has shown the safety and efficacy of halo-gravity traction as an operative adjunct. However, there are no reports specifically looking at halo-gravity traction in patients with skeletal dysplasia. Our purpose was to assess the safety and efficacy of traction in children with skeletal dysplasia who present with severe kyphoscoliosis.

**Methods:**

We retrospectively reviewed eight consecutive children with skeletal dysplasia who were treated with halo-gravity traction preoperatively. Six of the patients had a thoracoscopic anterior release prior to the halo-gravity traction. All patients were ambulatory and presented with severe, rigid kyphoscoliosis.

**Results:**

The mean duration of traction was 32 days. There were no neurologic complications with traction or after posterior spinal instrumentation. The majority of kyphoscoliosis correction was with the halo-gravity traction alone: major curve (MC) Cobb angle improved 41 %; C7–center sacral vertical line, 75 %; C7–MC apex, 21 %; and T2–T12 kyphosis, 35 %. Trunk height increased 37 % and thoracic height 44 %. An additional amount of correction was obtained with posterior spinal instrumentation (±fusion), decreasing MC Cobb angle an additional 23 %; C7–apex, 16 %; and T2–T12 kyphosis, 10 %. There was no additional correction of thoracic height. Two years after posterior spinal instrumentation (±fusion), a mild-to-moderate amount of correction was lost: MC Cobb angle decreased 23 %; compensatory Cobb angle, 28 %; C7–CSVL, 24 %; C7–S1, 22 %; regional kyphosis, 31 %; thoracic kyphosis, 29 %; and trunk height, 27 %.

**Conclusions:**

Among children with skeletal dysplasia and severe kyphosis, halo-gravity traction is well tolerated and safe. Most of the corrections in radiographic parameters were achieved with traction alone. Traction improves coronal balance, apical translation, thoracic height, and kyphosis. In this specific population, the potential for neurologic injury during corrective surgery is high. However, preoperative halo-gravity traction provides slow, progressive correction in a safe manner and avoided neurologic injury in these patients. This study did not compare patients without halo-gravity traction to patients with halo-gravity traction, therefore it cannot be concluded that going straight to instrumentation without traction will give a poorer radiographic result.

**Level of evidence:**

IV.

## Introduction

The management of rigid kyphoscoliosis in patients with skeletal dysplasia is not well discussed in the literature. There are several treatment options but no clear guidelines. The role of halo-gravity traction in skeletal dysplasia has never been examined.

The literature was reviewed using a MEDLINE search of the English-language literature and bibliographies of published manuscripts, and no articles on halo traction and skeletal dysplasia were found. Forty-two articles were found when searching keywords “kyphoscoliosis,” “skeletal dysplasia,” and “dwarfism,” and only four articles were relevant to the topic of kyphoscoliosis. All papers were descriptive case series (level IV evidence). There are limited resources on the management of kyphoscoliosis in the different forms of skeletal dysplasia [1–5].

In this population with rigid curves and poor bone quality, there are limited options for spinal instrumentation and correction. Furthermore, the potential for neurologic injury during corrective surgery is high in these patients [1, 2, 6]. The purpose of this study was to determine if slow correction with halo-gravity traction can be a safe, effective option for these large deformities in patients with skeletal dysplasia.

## Materials and methods

To determine the effectiveness and safety of halo-gravity traction in severe, rigid kyphoscoliosis, we retrospectively assessed a cohort of eight consecutive patients with skeletal dysplasia treated with halo-gravity traction. During this study period all skeletal dysplasia with severe kyphoscoliosis was treated with halo-gravity traction. All surgeries were performed by one surgeon at a single institution between 2006 and 2010 and had at least 2-year postsurgical follow-up. Inclusion criteria were skeletal dysplasia, preoperative halo-gravity traction, availability of a standing pretreatment and sequential traction radiographs, rigid curves with less than 25 % flexibility of the major curve Cobb angle, and thoracic kyphosis documented by lateral bending and extension radiographs over a bolster. Patients with traction performed only during surgery or pathology limited to the cervical spine were excluded. Eight patients satisfied these criteria and constituted the sample. Appropriate institutional review board approval was obtained for this study.

Subjects were assessed by age, sex, weight, height, and several radiographic parameters at date of halo-gravity traction application. Halo-traction-related complications and short- and long-term complications were documented. In each case, radiographs were repeated weekly while the patients were in traction. All traction radiographs were full-length, standing anteroposterior and lateral radiographs with the appropriate amount of gravity traction. Multiple radiographic parameters were calculated from each of the radiographs: major curve (MC) Cobb angle, C7 coronal plumb line to center sacral vertical line (C7–CSVL), C7 coronal plumb line to the MC apex (C7–MC apex), CSVL–MC apex, compensatory curve Cobb angle, trunk height, thoracic height, lumbar height, sagittal C7 plumb line–S1 (C7–S1), T2–T12 kyphosis, and regional kyphosis (the thoracic or thoracolumbar kyphosis measured over a short segment with the maximal amount of kyphosis).

All patients were diagnosed with a form of skeletal dysplasia by the treating surgeon and confirmed by a geneticist. The diagnoses were spondyloepiphyseal dysplasia (SED), spondyloendochondral dysplasia, camptomelic dysplasia (CD), spondylometaphyseal dysplasia (SMD), metatropic dysplasia (MD), spondyloepiphyseal metaphyseal dysplasia (SEMD), and Kniest syndrome (Table 1).

**Table 1:** Summary of descriptive characteristics

Patient	Diagnosis	Age (years)	Sex	Height (cm)	Weight (kg)	Anterior release?	Traction duration (days)
1	Kniest syndrome	9	M	102	20	Yes	23
2	Spondyloepimetaphyseal dysplasia	5	M	90	12	Yes	29
3	Metatropic dysplasia	7	M	102	21	Yes	14
4	Metatropic dysplasia	7	F	95	18	Yes	34
5	Spondylometaphyseal dysplasia	2	F	79	10	No	60
6	Spondyloepiphyseal dysplasia	9	M	71	13	No	23
7	Spondyloendochondral dysplasia	11	M	92	18	Yes	56
8	Camptomelic dysplasia	7	F	83	11	Yes	20

*M* male, *F* female

### Halo-gravity treatment protocols

All patients were brought to the operating room for halo placement. Normally, six to eight halo pins were placed to minimize the risk of loosening, as described by Mubarak et al. [7]. The pins were tightened appropriately, and proportionally to the patient’s age and size, and the overall density of the patient’s calvarium [10]. Traction was started with a low amount of weight (1.3–2.3 kg) usually immediately after the patient awoke. Traction was gradually increased at a rate of 1.0–1.5 kg per day as tolerated. The goal was to reach a maximum traction of 33–50 % of body weight. Traction was applied 24 h per day, with the weight decreased to 25 % of body weight when the patient was supine to prevent migration of the patient toward the head of the bed. Traction was applied while the patient was either in bed, a wheelchair, or a standing apparatus. During the traction interval, the patients continued to be ambulatory on a daily basis and also received daily respiratory treatments to optimize pulmonary function [10]. Patients had a nutrition consultation prior to admission and a nutrition consultation while admitted to optimize nutrition. Patients also received daily neurologic exams which included cranial nerve exams, strength and sensation in upper and lower extremities, and long tract signs. The length of the traction period was defined by the presence of radiographic evidence of curve improvement on weekly radiographs. Six of the patients had an anterior thorascopic spinal release the same day as application of halo traction. All the patients subsequently had posterior distraction-based instrumentation [growing rod; vertical expandable prosthetic titanium rib (VEPTR); or a definitive spinal fusion] within the same hospital admission. With consideration of the patient’s age, remaining growth, and the goal of maintaining spinal growth while controlling the deformity, the majority of the patients had VEPTR or growing rod procedures (Table 2). Only two patients had a posterior fusion after the traction and were older (Table 2). Once the posterior instrumentation was performed, the halo was removed in all the patients except two. Two patients wore the halo for an additional month to maintain a gentle corrective force with external immobilization. Both of these patients had severe kyphosis. The radiographic parameters were calculated after posterior instrumentation and at 2 years follow-up from instrumentation (Table 3).

**Table 2:** Summary of surgical procedures and radiographic parameters

Patient	Instrumentation procedure	Anterior release?	Traction duration (days)	Pre-op MC Cobb	Post traction	Post instrumentation
1	Posterior spinal fusion T3–L2	Yes	23	41°	22°	9°
2	Growing rod T2–L4	Yes	29	83°	48°	13°
3	VEPTR T3–L4	Yes	14	74°	52°	20°
4	VEPTR T3–L4	Yes	34	58°	47°	31°
5	Growing rod T1–L3	No	60	67°	21°	27°
6	VEPTR T3–L2	No	23	117°	74°	49°
7	Posterior spinal fusion T3–L4	Yes	56	81°	54°	27°
8	Growing rod T1–L3	Yes	20	72°	33°	36°

*Pre*-*op MC Cobb* major curve coronal Cobb angle prior to traction, *Post traction* MC Cobb angle after traction, *Post instrumentation* MC Cobb angle after posterior instrumentation

**Table 3:** Mean pre-traction, post-traction, and post-instrumentation parameters

	MC Cobb angle	Comp Cobb	C7–apex	Trunk ht	Thor ht	C7–S1	Regional kyphosis	T2–T12
Pre-traction	74°	55°	39 mm	185 mm	113 mm	63 mm	95°	94°
Post-traction	44°	29°	17 mm	254 mm	163 mm	48 mm	65°	61°
Post-instrumentation	27°	20°	11 mm	245 mm	158 mm	26 mm	50°	52°
Two-year follow-up	37°	30°	18 mm	229 mm	146 mm	35 mm	64°	64°
Traction % correction	41	48	21	37	44	24	32	35
Instr. % correction	23	15	50	^a^	^a^	34	16	10
Loss of correction at two-year follow-up (%)	23	28	23	27	27	22	31	29

*Post instrumentation* 1 month after posterior spinal instrumentation, *Two*-*year follow*-*up* 2 years after posterior spinal instrumentation, *Traction % correction* the percent correction with traction alone, *Instr. % correction* the additional percent correction 1 month after posterior spinal instrumentation compared to the post-traction values, *Loss of correction at two*-*year follow*-*up* the percent loss of correction at two-year follow-up, *C7*–*apex* the distance (mm) of the C7 coronal plumb line to the major curve apex, *Comp Cobb* compensatory Cobb angle, *ht* height, *thor* thoracic, *C7*–*S1* the distance (mm) from C7 sagittal plumb line to S1, *T2*–*T12* thoracic kyphosis from T2–T12

^a^No correction obtained

### Statistical analysis

The mean and standard deviation (SD) were used to summarize the continuous data after the normality assumptions were met, while the paired sample *t* test was used to assess the effect of traction on the radiographic parameters. However, where variables violated the normality assumption, the median and interquartile ranges (IQR) were used to provide the summary statistics. Likewise, the hypothesis testing involving the paired *t* test was performed using the non-parametric alternative, the Wilcoxon rank sum test, when the normality assumption was violated. All tests were two-tailed, with <5 % as the significance level. The STATA statistical software version 11.0 (STATACorp, College Station, TX, USA) was used to perform the analyses. In addition, to determine how the duration of traction predicted the outcomes, we used the simple linear regression model and obtained the regression equation. However, we performed a post-hoc power analysis using a type one error tolerance of 5 %, pre- and post-halo traction radiographic parameters (mean and SD), and found insufficient power for the regression model.

## Results

### Radiographic measurements

All eight patients met the criteria and had long-term radiographs. None of the patients had previous spinal procedures. Mean age was 7.1 years at time of halo traction. There were five males and three females (Table 1). The average curve magnitudes were as follows: major coronal curve magnitude, 74°; C7–CSVL, 22.8 mm; T2–T12 kyphosis, 94° (Table 3).

**Fig. 1:**
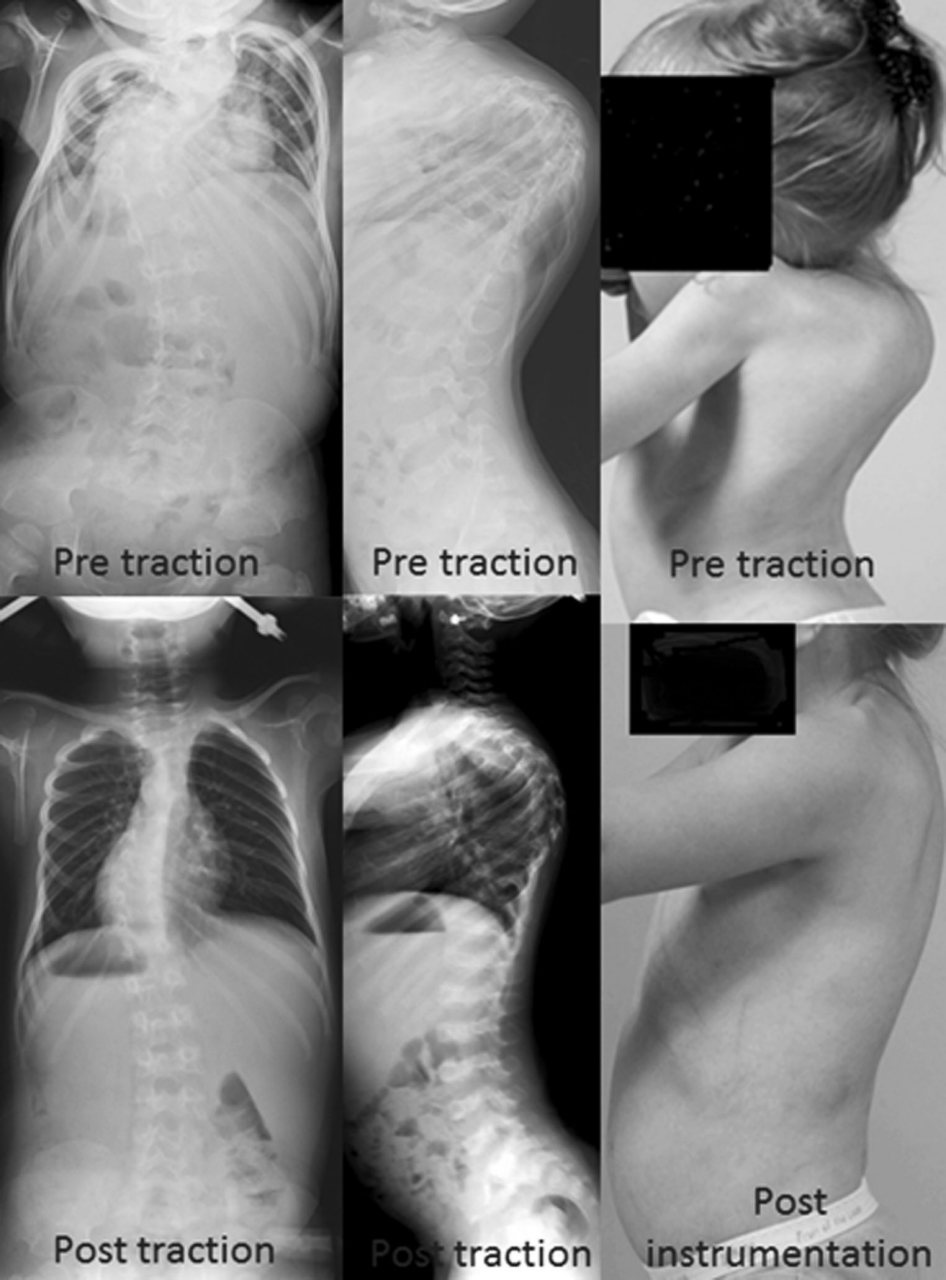
Thirty-month-old female with spondylometaphyseal dysplasia with a previous non-instrumented posterior spinal fusion at an outside institution presented with a rigid kyphoscoliosis. The patient was effectively treated with perioperative halo-gravity traction as shown

The duration of traction ranged from 2 to 8 weeks, with a mean duration of traction of 32 days. There were significant differences (*P* < 0.05) between pre-traction and post-traction MC Cobb angle, compensatory Cobb angle, C7–CSVL, C7–MC apex, CSVL–MC apex, trunk height, thoracic height, lumbar height, T2–T12 kyphosis, and regional kyphosis (Fig. 1). In contrast, there were no significant differences (*P* > 0.05) in CSVL–MC apex, lumbar lordosis, or sagittal C7–S1 plumbline (Table 3).

### Coronal plane parameters

After halo traction, the MC Cobb angle decreased 30° (−41 %, *P* = 0.009). Compensatory Cobb angle decreased 26° (−48 %, *P* = 0.004). There was a 17-mm improvement in global translation (C7–CSVL) toward the midline (75 % correction, *P* = 0.004). There was 21 mm of regional translation (C7–MC apex) of the apex toward the midline (55 % correction, *P* = 0.017) (Table 4).

**Table 4:** Correction with halo-gravity traction

Parameter	Mean correction	Percent correction	*t*	*P* value
MC Cobb	30°	41	3.0	*P* < 0.009
Comp Cobb	26°	48	3.7	*P* < 0.004
C7–apex (mm)	21	21	2.8	*P* < 0.017
Trunk height (mm)	68	37	−4.1	*P* < 0.001
Thoracic height (mm)	50	44	−3.0	*P* < 0.009
Regional kyphosis	30°	32	4.4	*P* < 0.001
Thoracic kyphosis	33°	35	4.6	*P* < 0.001

*MC Cobb* major curve Cobb angle, *comp Cobb* compensatory Cobb, *thoracic kyphosis* measured kyphosis from T2–T12, *C7*–*apex* the distance (mm) of the C7 coronal plumb line to the major curve apex, *t* (difference of the mean)/(standard error) from paired sample *t* test analysis

### Height parameters

The mean increase in trunk height was 68 mm (+37 %, *P* = 0.001). The mean increase in thoracic height (T1–T12) was 50 mm, meaning the thoracic height increased an impressive 44 % (*P* = 0.009) (Fig. 1). The mean increase in lumbar height (L1–S1) was 19 mm (+26 %, *P* = 0.036). Seventy-three percent of the improvement in trunk height was due to the increase in thoracic height (Table 4). One patient had a preoperative and a postoperative traction CT scan of the chest. The total lung volume expanded 107 %.

### Sagittal plane parameters

Most of the patients’ correction was in thoracic height and kyphosis. After halo traction, the thoracic kyphosis decreased 33° (−35 %, *P* = 0.001). The regional kyphosis decreased 30° (−32 %, *P* = 0.001) (Table 4), improving to a normal range for this patient population.

### Thoracoscopic anterior release

The effect of thoracoscopic anterior release with halo-gravity traction versus halo-gravity traction alone showed no statistically significant difference in the radiographic parameters (*P* > 0.05 for all parameters). However, we performed a post-hoc power analysis using a type one error tolerance of 5 %, thoracoscopic anterior release versus no release (mean and SD), and found insufficient power.

### Height, weight, age, and duration of traction as predictors of radiographic outcomes

Simple linear regression analysis was performed, using pre-traction height, weight, age, and duration of traction to predict the amount of correction in the radiographic parameters. However, a post-hoc power analysis using a type one error tolerance of 5 %, pre- and post-halo traction radiographic parameters (mean and SD), found insufficient power for the regression model. Therefore, these calculations have limited value. The patients’ height, weight, age, or duration of traction had no statistically significant relationship (*P* > 0.05) with the amount of correction in the MC Cobb angle (Table 5). Similarly, no statistically significant relationship was seen in the other radiographic parameters (*P* > 0.05 for all parameters).

**Table 5:** Linear regression analysis for MC Cobb angle

	β coefficient	*R* ^2^	*P* value
Height	−1.40	0.49	0.09
Weight	−2.04	0.16	0.32
Age	1.22	0.02	0.72
Duration of traction	−0.08	0.01	0.89

*MC Cobb* major curve Cobb, *R* ^*2*^ coefficient of determination

### The effect of posterior spinal instrumentation

Additional correction in the sagittal and coronal profile of the patient was obtained with definitive posterior spinal instrumentation: MC Cobb angle improved an additional 23 %; compensatory Cobb angle, 15 %; C7–apex, 16 %; C7–S1, 34 %; regional kyphosis, 16 %; and T2–T12 kyphosis, 10 %. However, with posterior spinal instrumentation, trunk height decreased 5 % and thoracic height 4 % from the amount of correction obtained with traction alone (Table 3).

### Two years follow-up

Two years after posterior spinal instrumentation (±fusion), a mild-to-moderate amount of correction was lost: MC Cobb angle decreased 23 %; compensatory Cobb angle, 28 %; C7–CSVL, 24 %; C7–apex, 23 %; C7–S1, 22 %; regional kyphosis, 31 %; thoracic kyphosis, 29 %; trunk height, 27 %; and thoracic height, 27 % (Table 3).

### Complications

No perioperative complications (neurologic, pulmonary, or other clinical issues) occurred through the entire period of halo-gravity traction, except one pin-tract infection. There were no cranial nerve or spinal cord deficits in this patient cohort. Halo-gravity traction was well tolerated. One patient suffered temporary paraplegia after the fusion procedure and was related to anterior graft extrusion that was later revised. Six months after the revision the patient had residual dorsiflexion weakness but could ambulate with bilateral ankle–foot orthoses and no other assistive devices. No postoperative wound infections were encountered in the entire cohort. One patient suffered proximal junctional kyphosis 3 months after the fusion but was managed without revision surgery and just observation. The patient’s kyphosis increased but stabilized.

## Discussion

This study was conducted to assess the safety and efficacy of halo-gravity traction in severe rigid kyphoscoliosis of skeletal dysplasia. Furthermore, the duration of traction and other prognostic factors such as weight, height, and age on the outcomes were evaluated. Halo-gravity traction was well tolerated with no complications in the study. Furthermore, there was significant improvement in the radiographic parameters. With the sample size, the role of duration on traction, height, weight, and age could not be defined.

While our study provides useful data for assessing the effectiveness and safety of traction in children with skeletal dysplasia, there are some limitations. First, we used a retrospective cohort design, which has a tendency of information and selection biases, thus influencing the internal validity of the findings. However, it is highly unlikely that our findings are driven solely by these biases, since we performed a thorough check to ensure the reliability of the data used in this study. Secondly, this study is underpowered, which limits our ability to utilize the duration of traction and other prognostic factors to predict the radiographic outcomes in our study; however, this is a very rare group of disorders, and without large, multicenter studies, a substantial number of patients would be difficult to assemble for analysis.

Only one case of the use of halo-gravity traction in skeletal dysplasia has been published. This paper described a single case of preoperative halo traction used in a patient with camptomelic dysplasia. Preoperatively, this patient had progressive respiratory distress that necessitated intubation. With halo traction, the surgeons were able to wean the patient from ventilator use and improve respiratory function [5, 9, 10].

When compared with other halo-gravity traction studies, similar coronal correction was obtained. In 19 scoliosis cases, Sink et al. [11] showed that with preoperative halo-gravity traction alone the major curve Cobb angle improved 35 %, global coronal imbalance improved 60 % with 18 mm of translation, and trunk height increased 53 mm. Our experience was similar. In the present study, the major curve Cobb angle improved 25 %, global coronal imbalance improved 75 % with 17 mm of translation, and trunk height increased 68.4 mm.

We surmise that some of the correction was due to the patients’ young age, inherent soft-tissue flexibility, and low body weight. Previous literature has not adequately compared the results of halo traction between early onset scoliosis and adolescent scoliosis [8, 11–15]. With the young age and low bulk mass of the paraspinal muscles in our patients, we obtained large corrections with halo-gravity correction despite rigid kyphoscoliotic deformities. A critique can be made that the patients have less strength and the bending films are not accurate measures of preoperative flexibility. However, all patients on examination preoperatively and in the operating room had rigid curves.

In our study, no correlation was seen between preoperative age, weight, or height with the measured (radiographic) correction of halo-gravity traction. This is most likely due to the small sample. One paper [15] demonstrated that the greatest correction was within the first week of halo-gravity traction. This was a very different population from ours, and the authors recommended 3 weeks of halo-gravity traction [14]. There are no other studies that provide recommendations on the length of halo-gravity traction. In Sink et al.’s study [11], the patients had a minimum 6 weeks of traction, and progressive correction was noted throughout. Their population matches our study’s patients in regard to age; in the other studies, the patients were older [1–5, 8–15]. No conclusions can be made on the optimal length of traction in skeletal dysplasia due to the sample size. Our recommendation, based on the data, is weekly radiographs, which are helpful in determining the response of traction and the leveling off of correction.

Another major area of correction in the current study was in thoracic kyphosis. All patients had severe, rigid kyphosis (93.6°), and a 33 % correction was obtained. None of the preoperative halo-gravity traction studies have shown such thoracic kyphosis correction with only halo-gravity traction [8, 14, 15]. This is important because control of kyphosis may prevent implant failure and/or proximal junctional kyphosis after spinal instrumentation.

In this study, thoracoscopic anterior release did not show statistically significant correction when compared to halo-gravity traction alone. Though anterior release may affect deformity correction, our sample size was not large enough to provide a stable finding. Similarly, in the Watanabe et al. [15] study of 21 patients with scoliosis treated with halo-gravity traction alone or in combination with an anterior release, there were similar results between the two groups. Perhaps selection bias to stiffer curves requiring anterior release would skew any analysis in a retrospective study.

Considerable correction in thoracic height was obtained with halo-gravity traction alone (44 % increase). Based on this fact, it can be assumed that halo traction also optimizes lung volumes by an increase in the thoracic height. This translated into one patient more than doubling his lung volume with a trial of halo-gravity traction.

Among children with skeletal dysplasia and severe kyphosis in this study, halo-gravity traction was well tolerated and safe. In Bridwell et al.’s [8] review of 33 children with severe kyphoscoliosis treated with halo-gravity traction there were two temporary neurologic complications (brachial plexus and triceps palsy) with no permanent neurologic complications. Most of the corrections in radiographic parameters were achieved with traction alone. Traction improves coronal balance, Cobb angle of the major curve, apical translation, and, particularly, thoracic height and kyphosis. In this specific population with skeletal dysplasia and severe kyphoscoliosis, the potential for neurologic injury during corrective surgery is high. However, preoperative halo-gravity traction provides slow, progressive correction in a safe manner, and it did not cause neurologic injury in these patients.
